# A survey describing the use of complementary therapies and medicines by women attending a family planning clinic

**DOI:** 10.1186/1472-6882-13-224

**Published:** 2013-09-11

**Authors:** Caroline A Smith, Deborah J Bateson, Edith Weisberg

**Affiliations:** 1 Centre for Complementary Medicine Research, University of Western Sydney, Penrith, Australia; 2 Family Planning New South Wales, Sydney, Australia

**Keywords:** Complementary therapies and medicines, Women’s health, Reproductive health, Family planning, Survey

## Abstract

**Background:**

Complementary medicines (CMs) are widely used by women. Although, women in Australia are frequent users of CM, few studies have examined their utilisation by women attending a family planning service. The aim of this study was to examine (i) the extent of and type of CM, (ii) women’s views about safety and efficacy, and (iii) the factors influencing women’s decision-making.

**Methods:**

A cross-sectional survey using a convenience sample of 221women aged greater than 18 years attending a family planning (FP) service was undertaken over a two week period in Sydney, Australia. An anonymous self-administered questionnaire was designed to examine women’s current and previous use of CMs, their attitudes towards safety and effectiveness, the factors influencing their decision-making, and their disclosure of CM use to a FP health professional. Demographic questions were designed to describe the diversity of the participants. Logistic regression was used to examine the association between CM use and demographics.

**Results:**

Sixty-seven percent of women surveyed were currently using CMs, and 83% reported use during the previous 12 months. Most respondents utilised CMs to maintain their general health or for prevention of ill health. Over 30% of women lacked information to make an informed response to questions examining their views about the safety of CMs. Forty-four percent of participants stated they discussed their use of CMs with their FP providers. The main reason why women did not mention CMs was they did not see the relevance to their consultation (43%). Lower rates of CM use were found for younger women (OR 0.24, 95% CI 0.09-0.61), and those not completing high school (OR 0.44, 95% 0.20-1.00).

**Conclusion:**

The use of CM is very common among women attending an Australian FP clinic, however our findings may not be generalisable to all women. We identified a notable gap in women’s awareness of the potential for interactions between CM and prescribed medication. Our findings also emphasise the need for healthcare providers to initiate discussions with clients about their utilisation of CM.

## Background

The use of CMs among women seeking reproductive health care in clinical settings in relation to menopause [1], infertility [2], and pregnancy [3], has been investigated. A review of this literature indicates that resorting to some form of CM for fertility enhancement varies from 29% to 91% [2]. The Australian Longitudinal Study on Women’s Health in 2006 revealed that 81% of pregnant women reported the self-prescribed use of vitamins and minerals, 33% consulted with a CM practitioner, and 19% consumed herbal medicines [3], and nearly one in six women in the United States reported having taken a herbal supplement in 2000 [4].

Motivations for CM use among women are little understood. However CM use during pregnancy is driven by women’s motivations to take an active role in the maintenance of their health [5], a belief in a holistic approach to health and treatment [6], and/or an interest in ‘natural therapies’ that have fewer side effects [3,5,7,8].

Women view CMs as safe and as at least equally efficacious as medical prescriptions [9,10]. However, safety concerns arise from the concurrent use of conventional medical treatment with CM therapy [11] and poor communication about CM consumption between patients and their health care providers. A lack of disclosure of CM consumption to conventional health practitioners is not uncommon [4] and, consequently, interactions with medications and herbal therapies do occur. In the area of family planning (FP), which encompasses sexual and reproductive health, concerns exist for example about an interaction between St John’s wort (*Hypericum perforatum*) with certain hormonal contraceptives [12]. Case reports of oral contraceptive (OC) failure coinciding with use of St John’s wort are on record and some studies have identified potential interactions [13,14]. A pilot study evaluating the effect of St John’s wort on OC therapy brought to light a link between the resulting increased metabolism of norethindrone and ethinylestradiol, and the occurrence of breakthrough bleeding, follicular growth, and ovulation [15]. These findings suggest St John’s wort may reduce the effectiveness of hormonal contraceptives, with the exception of depot medroxyprogesterone acetate injections and the levonorgestrel releasing-intrauterine system, whose efficacy does not appear to be impaired by the enhancement of the steroid metabolism in the liver. These findings are reflected in internationally recognised guidelines for the safe and effective provision of contraceptive methods [16].

Although, according to the literature, women in Australia are frequent users of CMs [2,3,5], few studies have examined their utilisation by women attending a FP service. Understanding women’s use and extent of CM utilisation, patterns and motivations for use, and disclosure of CM in this clinical setting is important with assisting health care providers to promote safe and effective contraception. The aim of this study was to examine (i) the extent of and type of CM, (ii) women’s views about safety and efficacy, and (iii) the factors influencing women’s decision-making.

## Methods

We undertook a cross-sectional survey of women attending the main metropolitan urban Family Planning New South Wales (FPNSW) clinic at Ashfield in Sydney. Ethics approval was obtained from the Family Planning New South Wales Ethics Committee. Women were eligible to participate if they were 18 years of age or older, and were able to read and write English. Eligibility was confirmed from clinical notes.

An anonymous self-administered questionnaire was designed to examine previous use of CM (defined as use in the previous 12 months), and women’s current use (defined as at present and ongoing) their attitudes towards safety and effectiveness, the factors influencing their decision-making, and their disclosure of CM use to a FP health professional, women attending for the first time were asked not to answer this question. Demographic questions were designed to describe the diversity of the participants including age, education, country of birth and languages spoken at home. Questions about CM use were taken from a previously published survey [10]. This questionnaire included a broad and inclusive definition of CM medicines and therapies, and distinguished between vitamins and mineral supplements prescribed or not prescribed by a doctor. This distinction reflects the regulatory framework in Australia where complementary medicines are regulated as medicines under the Therapeutic Goods Act1989 [17]. A review of other surveys was also undertaken and included those previously used by the author (CS) [5,18,19]. Consultations were also held with the medical and research directors and the research team at FPNSW to discuss content and finalise the design. CM treatments included herbal medicines, vitamin and mineral supplements, other nutritional supplements, traditional medicines such as traditional Chinese medicines, homoeopathic medicines, and aromatherapy oils (where they make therapeutic claims).

The questionnaire was piloted on ten women attending the clinic in early January 2011. After these interviews it was apparent that minor modifications to the wording of some questions were required. We did not undertake a power calculation for the sample size; instead we decided to survey all women attending the clinic who met the eligibility criteria during a two-week period in January, 2011. It was considered that this time period would generate a population that would be representative of the total population. All clinic sessions were attended by the research assistant. Women were approached by a research assistant and invited to participate in the survey. Women were informed that their participation was voluntary. Only verbal consent was obtained as completion of the self-administered questionnaire by women before or after their consultation confirmed consent.

Statistical analyses were carried out using SPSS software (the IBM Statistical Package for the Social Sciences 19, Chicago, IL). Descriptive statistics, using frequencies and percentages were calculated for demographic and categorical data, and chi-squared tests were undertaken to compare CM patterns of use with socio-demographic characteristics. We subsequently ran logistic regression models to examine the association between CM use, attitudinal data and demographics. For the nominal regression model attitude (dependent vaiable) was re-coded to three categories; agree, disagree and don’t know. The co-variants included age and education, and education was re-coded to tertiary educated or not. We report on adjusted odds ratios and a 95% confidence interval. The conventional 0.05 level of significance was used to test for statistical differences.

## Results

Between 11 January and 24 January 2011, 221 questionnaires were distributed to eligible women. Of this convenience sample, 209 who agreed to participate in the study completed the questionnaire (94% response rate). Twelve women declined. The sample represented a varied population which spanned all eligible reproductive age groups (Table 1). The majority of women were tertiary educated. Most were born in Australia but other nationalities were well represented, with women born in 33 other countries, who between them spoke 11 languages other than English at home. Comparing the characteristics of our study population with the profile of clients attending the Ashfield Clinic at FPNSW, we found our sample had a similar age profile and country of birth to the women attending the Ashfield clinic, however our study population was represented by a greater proportion of women who spoke only English at home (p = 0.01), and whose education profile differed (p < 0.01). Ashfield is one of the most culturally diverse communities in inner Sydney with 43% of the population born overseas.

**Table 1:** Characteristics of women participating in the survey and women attending the clinic

		**Women participating in the survey (n = 209)**			**All women attending Ashfield clinic in 2011 (n = 6242)**			***p*-value**
		**n**	**%**	**(95% CI)**	**n**	**%**	**(95% CI)**	
Age								
	18–24 years	43	20.5	(15.3-26.7)	946	15.1	(14.2-16.0)	p = 0.113
	25–34 years	56	26.7	(20.9-33.3)	1907	30.5	(29.4-31.7)	
	35–44 years	48	22.9	(17.4-29.2)	1629	26.1	(25.0-27.2)	
	45 + years	54	25.8	(20.0-32.3)	1760	28.2	(27.0-29.3)	
	Missing data	8	3.8	(1.6-7.4)				
Highest level of education								
	Did not finish high-school	8	3.8	(1.6-7.4)				
	Finished high-school*	32	15.3	(10.7-20.9)	1850	29.6	(28.5-30.7)	p < 0.001
	TAFE or similar#	52	24.8	(19.1-31.3)	1368	21.9	(20.9-22.9)	
	University degree	108	51.6	(44.6-58.6)	2962	47.4	(46.2-48.7)	
	Missing data	9	4.3	(1.9-8.0)				
Country of birth								
	Australia	130	62.2	(55.2-68.8)	3616	57.9	(56.6-59.1)	p = 0.218
	United Kingdom	9	4.3	(1.9-8.0)	328	5.2	(4.7-5.8)	
	New Zealand	6	2.8	(1.0-6.1)	186	2.9	(2.5-3.4)	
	China	6	2.8	(1.0-6.1)	405	6.4	(5.9-7.1)	
	Other	49	23.4	(17.8-29.7)	1707	27.3	(26.2-28.4)	
	Missing data	9	4.3	(1.9-8.0)				
Language spoken at home								
	English	183	87.5	(82.3-91.7)	5297	84.8	(83.9-85.7)	p = 0.014
	Other	18	8.6	(5.1-13.2)	945	15.1	(14.2-16.0)	
	Missing data	8	3.8	(1.6-7.4)				

*Finished high school only; #TAFE, Technical and further education institute.

Due to rounding not all percentages add up to 100%.

### Patterns of CM use

Eighty-three percent (95% CI 78.0-88.4) of women reported they had used CM products within the previous 12 months, and 67% (95% CI 60.6-73.7) of the sample indicated they were currently using CM products (Table 2). The most popular CM products included vitamins and minerals. Consultations with complementary therapists in the previous 12 months were common, with 70 (33%), 95% CI 27.1-40.3) women reporting visits; a smaller number of women were currently seeing a CM practitioner (Table 2).

**Table 2:** Use of complementary medicine and therapies (CM) by women

***Complementary medicines n = 209***	**Complementary medicines use in previous 12 months**			**Currently using complementary medicines**		
	**n**	**%**	**(95% CI)**	**n**	**%**	**(95% CI)**
Any use of CM	175	83.7	(78.0-88.4)	141	67.4	(60.6-73.7)
Vitamins not prescribed by a doctor	117	55.9	(48.9-62.8)	79	37.8	(31.2-44.7)
Vitamins prescribed by a doctor	32	15.3	(10.7-20.9)	20	9.5	(5.9-14.3)
Mineral supplements not prescribed by a doctor (e.g., zinc, calcium, selenium)	73	34.9	(28.4-41.8)	50	23.9	(18.3-30.2)
Mineral supplements prescribed by a doctor (e.g., zinc, calcium, selenium)	36	17.2	(12.3-23.0)	11	5.2	(2.6-9.2)
Other natural medicines from fish or animals (e.g., fish oils)	73	34.9	(28.4-41.8)	40	19.1	(14.0-25.1)
Herbal teas for health reasons (e.g., valerian, peppermint)	72	34,4	(28.0-41.3)	45	21.5	(16.1-27.7)
Other herbal medicine (e.g., evening primrose oil, black cohosh)	40	19.1	(14.0-25.1)	19	9.0	(5.5-13.8)
Chinese traditional medicines (e.g., Chinese herbs)	22	10.5	(6.7-15.5)	9	4.3	(1.9-8.0)
Aromatherapy oils for medicinal purposes	22	10.5	(6.7-15.5)	7	3.3	(1.3-6.7)
Homoeopathic medicines	15	7.1	(4.0-11.5)	8	3.8	(1.6-7.4)
**Consultations with complementary medicine practitioner**	**Consultation with complementary medicine practitioner in previous 12 months**			**Current consultation with a complementary medicine practitioner**		
Any consultation	70	33.5	(27.1-40.3)	39	18.6	(13.6-24.6)
Chiropractor	26	12.4	(8.2-17.6)	12	5.7	(3.0-9.8)
Acupuncturist	23	11.0	(7.0-16.1)	8	3.8	(1.6-7.4)
Naturopath	20	9.5	(5.9-14.3)	8	3.8	(1.6-7.4)
TCM practitioner	14	6.7	(3.7-10.9)	5	2.3	(0.7-5.4)
Osteopath	12	5.7	(3.0-9.8)	7	3.3	(1.3-6.7)
Other (e.g., reflexologist)	21	10.0	(6.3-14.9)	11	5.2	(2.6-9.2)

Categories are not mutually exclusive; women may have been using more than one product/modality. *CI* confidence interval, *TCM* traditional Chinese medicine.

The majority of women consulted with a CM practitioner or took CMs to maintain their general health, for prevention or to support their immune system, for instance to prevent a cold (Table 3). The most common specific health condition treated by CM related to depression and anxiety.

**Table 3:** Current and previous complementary medicine (CM) use by health condition

**Area of health n = 209**	**Use in previous 12 months**			**Current use**		
	**n**	**%**	**(95% CI)**	**n**	**%**	**(95% CI)**
General health (e.g., to stay healthy)	72	34.4	(28.0-41.3)	61	29.1	(23.2-35.8)
Preventative care (e.g., prevent a cold)	36	17.2	(12.3-23.0)	18	8.6	(5.1-13.2)
Support immune system	29	13.8	(9.4-19.3)	18	8.6	(5.1-13.2)
Depression/anxiety	21	10.0	(6.3-15.1)	14	6.7	(3.7-10.9)
Dysmenorrhoea	14	6.7	(3.7-10.9)	8	3.8	(1.60-7.4)
Sleeping problems/ insomnia	16	7.6	(4.4-12.1)	7	3.3	(1.3-7.6)
Premenstrual symptoms	13	6.2	(3.3-10.6)	10	4.7	(2.3-8.6)
Skin condition	12	5.7	(3.0-9.8)	6	2.8	(1.0-6.1)
Menopausal symptoms	10	4.7	(2.3-8.6)	8	3.8	(1.6-7.4)
Others (e.g., back pain, neck pain, stress, digestion, cramps, cystitis, subfertility, arthritis)	63	30.1	(24.0-36.8)	31	14.8	(10.3-20.3)

Categories are not mutually exclusive; women may have been using CM for more than one area of their health.

CI, confidence interval.

Thirty-four women never utilised CMs. Their reasons included: no need (n = 27), cost (n = 17), doctor or nurse has not recommended it (n = 5), lack of information about therapies available (n = 4), lack of information about safety (n = 3), or no interest (n = 1).

Our logistic regression analysis show a significant relationship between young women reporting less previous use (OR 0.23, 95% CI 0.07-0.72, *p* = 0.01) and current CM use (OR 0.24, 95% 0.09-0.61, *p* = 0.03). We also found that those not completing high school education were less likely to utilise CM (OR 0.39, 95% 0.15-1.02, *p* = 0.05). We found no relationship between CM use and the language spoken at home (Table 4).

**Table 4:** Logistic model of complementary medicine use among women by demographic variables

**Independent variables**		**Dependent variables**					
		**Previous CM use**			**Current CM use**		
		**OR**	**(95% CI)**	***p*-value**	**OR**	**(95% CI)**	***p*-value**
*Model 1: Age*							
	18-24	0.23	(0.07, 0.72)	0.01	0.24	(0.09, 0.61)	0.03
	25-34	0.85	(0.24, 2.96)	0.79	0.52	(0.20, 1.31)	0.16
	35-44	0.51	(0.15, 1.68)	0.26	0.34	(0.13, 0.88)	0.02
	45+	reference			reference		
*Model 2: Education*							
	Did not finish high school	0.39	(0.15, 1.02)	0.05	0.44	(0.20, 1.00)	0.05
	Finished high school	0.68	(0.74, 6.32)	0.73	0.70	(0.12, 4.08)	0.70
	TAFE or similar	0.65	(0.26, 1.64)	0.36	0.66	(0.32, 1.36)	0.27
	University degree	reference			reference		
*Model 3: Language spoken at home*							
	English	1.5	(0.48, 5.15)	0.44	1.08	(0.38, 3.04)	0.87
	Other	reference			reference		

*OR* Odds Ratio, *CI* confidence interval, *TAFE* Technical and further education institute.

### Attitudes towards safety and effectiveness of CM

Women’s responses about the safety of CM indicate that many may lack information to make an informed response to these statements; a third of them selected a ‘don’t know’ answer (Table 5).

**Table 5:** Survey participants’ beliefs and views about complementary medicines

**n = 209**	**Strongly disagree**			**Disagree**			**Agree**			**Strongly agree**			**Don’t know**		
	**n**	**%**	**95% CI**	**n**	**%**	**95% CI**	**n**	**%**	**95% CI**	**n**	**%**	**95% CI**	**n**	**%**	**95% CI**
**I only use**															
**CM as a last resort**	42	20.1	14.8-26.1	84	40.1	33.4-47.1	25	11.9	7.8-17.1	2	0.9	0.12-3.4	45	21.5	16.1-27.7
**CMs are not as risky as prescribed medication**	11	5.2	2.6-9.2	62	29.6	23.5-36.3	67	32.0	25.7-38.8	17	8.1	4.8-12.7	37	17.7	12.7-23.5
**CMs can cause dangerous side effects**	19	9.0	5.5-13.8	60	28.7	22.6-35.3	45	21.3	16.1-27.7	9	4.3	1.9-8.0	61	29.1	23.1-35.8
**Ingredients in CM make them safe to use**	11	5.2	2.6-9.2	40	19.1	14.0-25.1	68	32.5	26.2-39.3	7	3.3	1.3-6.7	69	33.0	26.6-39.8

Percentages may not add up to 100% due to rounding.

Using a multinominal logistic regression we explored the association between attitudes towards safety and effectiveness (dependent variables), and age and education (covariants). Based on our findings in Table 4 the demographics of age and education were selected to examine if they would predict the direction of women’s attitudes. We found no significant individual effects of age and education on attitudes (Table 6).

**Table 6:** Multinominal regression of participants’ beliefs and views about complementary medicines by demographic variables

**n = 209**		**OR**	**95% CI**
**I only use CM as a last resort**^**a**^			
*Disagree*			
Age	18–24	1.60	(0.43-6.0)
	25–34	2.68	(0.58-12.4)
	35–44	1.38	(0.34-5.63)
	45+	0^b^	
Tertiary Education			
	No	3.26	(0.80-13.3)
	Yes	0^b^	
*Don’t know*			
Age	18–24	0.52	(0.16-1.61)
	25–34	2.11	(0.58-7.67)
	35–44	0.91	(0.29-2.83)
	45+	0^b^	
Tertiary Education			
	No	1.81	(0.48-6.8)
	Yes	0^b^	
**CMs are not as risky as prescribed medication**			
*Disagree*			
Age	18–24	2.59	(0.84-8.0)
	25–34	0.68	(0.21-2.1)
	35–44	1.05-	(0.33-3.35)
	45+	0^b^	
Tertiary Education			
	No	1.08	(0.37-3.15)
	Yes	0^b^	
*Don’t know*			
Age	18–24	1.05	(0.40-2.77)
	25–34	0.61	(0.26-1.43)
	35–44	0.77	(0.31-1.89)
	45+	0^b^	
Tertiary Education			
	No	1.3	(0.55-3.14)
	Yes	0^b^	
**CMs can cause dangerous side effects**			
*Disagree*			
Age	18–24	1.73	(0.59-5.03)
	25–34	0.81	(0.29-2.24)
	35–44	0.75	(0.26-2.09)
	45+	0^b^	
Tertiary Education			
	No	0.81	(0.31-2.08)
	Yes	0^b^	
*Don’t know*			
Age	18–24	1.70	(0.57-5.04)
	25–34	1.56	(0.60-4.06)
	35–44	1.17	(0.43-3.12)
	45+	0^b^	
Tertiary Education			
	No	0.81	(0.21-1.49)
	Yes	0^b^	
**Ingredients in CM make them safe to use**			
*Disagree*			
Age	18–24	1.72	(0.64-4.6)
	25–34	0.43	(0.17-1.1)
	35–44	0.79	(0.30-2.1)
	45+	0^b^	
Tertiary Education			
	No	1.28	(0.49-3.38)
	Yes	0^b^	
*Don’t know*			
Age	18–24	0.74	(0.23-2.37)
	25–34	0.47	(0.17-1.29)
	35–44	1.11	(0.30-2.10)
	45+	0^b^	
Tertiary Education			
	No	1.99	(0.73-5.41)
	Yes	0^b^	

the reference category is agree.

b this parameter is set to zero because it is redundant.

OR, Odds Ratio, CI, confidence interval; CM complementary medicine.

### Views about CM and reasons for use

Forty-nine percent of women considered CM as being a ‘*more natural*’ benefit. Less frequently mentioned advantages of CM utilisation included ‘*better than taking drugs*’ (44%), ‘*can complement existing health care*’ (40%), ‘*effective, seems to work*’ (38%), ‘*offers a form of self-care*’ (36%), ‘*has a focus on prevention*’ (35%), ‘*a lack of side effects*’ (29%), and *‘is perceived to be safe*’ (21%). The disadvantages of CMs were viewed as being ‘*expensive*’ (37%), ‘*a lack of research on how it works*’ (28%), and ‘*uncertain if it will work*’ (26%).

### Information and communication

Information on CM was mainly obtained from family and friends (50%), the internet (49%), printed material (books and magazines 49%), health professionals (38%), and CM practitioners (25%).

Women who had used CM in the previous 12 months were asked if they discussed their self prescribing use of CM or consultation with a CM practitioner during their FP consultation. Forty-four percent of participants had; those who had not indicated that they considered CM unrelated to consultation at FPNSW (43%), did not view CM as a medicine (30%) or were worried about discussing this with their doctor or nurse (5%). Two women explained in an open response that they had not mentioned it because they were not asked.

## Discussion

Over 80% of women attending the metropolitan Family Planning New South Wales (FPNSW) clinic at Ashfield we surveyed had visited a CM practitioner or used CMs in the previous 12 months, and two thirds were currently using CMs. Our study highlights the popularity of dietary supplements, including vitamins and minerals, and consultation with chiropractors and acupuncturists. This population reported using CM for health maintenance and self care. The advantages of CMs identified by participants included enhanced self-care, perceived safety, and being ‘natural’. Most participants did not mention their CM use because they considered this information irrelevant to their clinical consultation (rather than out of a fear of rejection); because they had not been asked; or because this information had been dismissed after disclosure.

Although the prevalence of CM use we recorded was slightly higher than that (74.4%, 95% CI 70.7–78.1) reported in the last national survey undertaken in Australia [20], it is consistent with that observed by other authors among pregnant Australian women [3]. A cohort study has shown that women who consulted with CM practitioners had increased over time, with 9%, 16%, 21% and 33% of women consulting a CM practitioner in 1996, 2000, 2003, and 2006, respectively [3] The slightly higher rate of CM utilisation in our study may be explained by the fact that women attending a FP service may be more likely to use CM for self care or consult with practitioners, or our definition of CMs.

Women accessed information on CMs most frequently from non-medical sources including the internet, family and friends. These findings are supported by other studies from different clinical populations [20]. Forty-four percent of clients mentioned their consultations and self-prescribed use of CM to their healthcare provider. There are no reported studies of a general clinic population with which to compare our findings. However a study of pregnant women in the USA suggests similar levels of non-disclosure (52%) to health care providers [21].

We have shown that many women combine conventional health care with self-administered CM and consultations with CM practitioners. The findings also highlight that some women have limited awareness of the importance of disclosing CM use to the practitioner in the FP unit. This finding is important because the latter may, therefore, not receive full information to include in the clinical history of the patient or become aware of a potential interaction between prescribed medications and CM remedies.

The survey had a response rate of 94%, reducing the potential for a response-bias. Also the opportunity for measurement error was reduced by (i) piloting of the questionnaire, (ii) revising the instrument prior to implementation of the study, and (iii) having a researcher on site to assist with enquiries.

However, there are limitations to the study. Respondents were self-selected rather than randomly chosen, and therefore may not be representative of the clinic population as a whole. In fact we know that the sample was not representative of the larger clinic population with regard to two socio-demographic characteristics, significantly more women in the sample spoke English at home, and had a different level of education attainment than the FPNSW clinic population. Compared with national socio-demographic data the FPNSW clinic population has a slightly higher (5%) overseas born population, with a higher preference for other languages at home (5%). Our sample maybe more comparable to all family planning organisations in Australia. In addition, because we undertook a snapshot of clinic attendees during a two-week period, bias may have been introduced if there were groups of women who did not attend at this time of the year.

The questionnaire included CM modalities reporting a therapeutic claim. Our criteria may have led to some modalities being excluded, and consequently to the finding of a lower rate of utilisation than would otherwise have been the case. There is no widely accepted operational definition of CM. Wiedland [22] and colleagues report on an operational definition for CM therapies based on (i) therapies that rely upon non-allopathic models of health, (ii) exclusion from standard treatment within the dominant medical system, and (iii) self-care or care delivered by alternative practitioners. A lack of efficacy was not a criterion for identifying CM. If we had adopted the definition of CM described by Wiedland and co-authors’, the reporting of CM use in our survey would likely to have increased. Despite these limitations, the similarities of our findings with national surveys support the validity of the study. Our results indicate that CM is widely used by women attending a FP service at an inner city clinic in Sydney.

Despite the increased awareness from research on the importance of health care providers discussing CM use with their patients, we highlight our findings in the context of a gap in knowledge in this clinical setting. Our findings also have implications for international FP programmes where use of traditional medicines (traditional Chinese medicine, Indian Ayurveda, and practices that include herbal medicines) are prevalent [23]. Women need to be informed that communication about use of CMs is relevant and important. Providing information and education to clinicians about what CMs are available and their benefits and risks would assist with demonstrating respect for women’s use of CMs. The study has drawn attention to non disclosure of CM in this clinic and the need for clinicians to be further informed about the potential for herb-drug interactions, and to be more proactive with seeking information about CM use, particularly among women using oral contraceptives. In our study 16 women reported use of St Johns wort in the previous 12 months, and six women were currently using this herb for anxiety of depression. Due to the anonymous nature of the questionnaire we do not know if these women were using oral contraceptives, however these is the possibility of potential a herb-drug interaction arising for some women. Further discussion about CMs may also have a positive impact on satisfaction with the consultation as clients may feel they are being listened to and that their healthcare providers are interested in their personal situation.

A large number of women appear to be uninformed about the safety of CM. We did not explore the reasons for women’s views towards the safety and effectiveness of CM, and this appears to be an under-researched area. Future research using qualitative designs would contribute valuable information to assist the development of appropriate communication strategies.

## Conclusion

We have demonstrated in our study the high frequency of CM utilisation for self-care among women attending an Australian FP clinic, however our findings may not be generalisable to all groups of women attending this clinic. We also identified a notable gap in women’s awareness of the risks and benefits of CM, the potential for interactions between CM and prescribed medication, and the importance of disclosure and communication of their consultations with CM practitioners. Other healthcare providers (e.g., family physicians) who may interact with this group of women should be aware of the widespread and frequent use of CM. Our findings also emphasise the need for healthcare providers to initiate discussions with clients about their utilisation of CM.

## Competing interests

The authors declare they have no competing interests.

## Authors’ contributions

CAS participated in the design, analysis, interpretation of the data, and drafted the manuscript. DB participated in the design and drafting of the manuscript. EW participated in the design and drafting of the manuscript. All authors read and approved the final manuscript.

## Pre-publication history

The pre-publication history for this paper can be accessed here:

http://www.biomedcentral.com/1472-6882/13/224/prepub
